# The Impact of Integrating a Parkinson's Specialist Psychiatrist Into the Multidisciplinary Team on Patients With Parkinson's and Cognitive Impairment

**DOI:** 10.1155/padi/6636111

**Published:** 2025-09-01

**Authors:** Ellen Tullo, Gayathri Rajesh Nair, Sarah Henry

**Affiliations:** ^1^Northumbria Healthcare NHS Foundation Trust, Newcastle Upon Tyne, UK; ^2^University of Sunderland Medical School, Sunderland, UK

**Keywords:** cognitive impairment, multidisciplinary team, Parkinson's disease, psychiatry

## Abstract

Cognitive impairment in Parkinson's disease (PD) is common, but there is scarce evidence as to how this group of patients can be most effectively assessed and managed. Our quality improvement project evaluated the impact of integrating a PD specialist psychiatrist (PDSP) into an existing multidisciplinary team (MDT) to allow direct referral of patients with cognitive impairment rather than to a separate service. We collected data over 1 year to map the referral trajectories of patients through the new pathway and estimated cost savings by comparison with the previous pathway. Eighty-five patients were referred to our PDSP, 47 with cognitive impairment. Estimated cost savings attributed to the new pathway were more than £1000, with the greatest savings associated with patients diagnosed with mild cognitive impairment (MCI). Integration of a PDSP into our MDT led to a more streamlined service, rapid access to diagnosis and management and likely cost savings.

## 1. Introduction

Parkinson's disease (PD) is traditionally recognised by the motor symptoms of tremor, rigidity and bradykinesia (slowness of movement). However, recent clinical studies have confirmed the high frequency of a wide range of “nonmotor” symptoms that may precede the classic motor symptoms. Nonmotor symptoms can affect multiple body systems and include psychiatric symptoms such as depression, anxiety, hallucination and memory problems, which have a significant impact on patients' quality of life (QoL) [1].

Epidemiological studies suggest that symptoms of depression occur in 40%–50% of patients with PD [2] and anxiety in 20%–50% [3]. Mood disorders may arise due to the impact of motor symptoms on patients' QoL, but may also occur as a result of the same pathological brain changes that cause motor symptoms, and may fluctuate in parallel with motor symptoms [4]. Hallucinations are also common, with 75% of patients affected at some point in the course of their disease and are associated with cognitive decline and mortality [5]. Treatment of hallucinations or other symptoms of psychosis is challenging due to the risks related to antipsychotics, and there are no standardised guidelines [6]. PD increases the risk of developing memory problems, and approximately 20% of patients will have a degree of cognitive impairment at the time of diagnosis [7]. The risk of dementia increases with the duration of PD, with a lifetime risk of 80% [8]. Psychiatric symptoms are implicated in the majority of unplanned admissions of patients with PD to hospital, which have significant associated costs and risks to QoL [9]. Symptoms associated with dementia are a common cause for admission and are 11 times more likely in patients with PD than those without between the ages of 50–69 [10].

Multidisciplinary team (MDT) care for PD is known to be effective in improving QoL [11]; however, there are no specific guidelines around the optimum diagnostic and management pathway for patients with PD and psychiatric symptoms such as cognitive impairment. Psychiatric symptoms in PD are complex and may require the expertise of psychiatry and psychology to adequately diagnose, treat and monitor. It is particularly challenging to accurately diagnose dementia with Lewy bodies (DLB) in the early stages of the disease process, even for PD specialists [12].

Our PD service at a district general hospital in Northeast England has a well-established MDT comprising specialist nurses, geriatricians, physiotherapists and speech and language specialists, operating across a large geographical area. Patients with PD and cognitive impairment were previously referred to a different service for assessment, diagnosis, treatment and monitoring. Our aim was to adapt our existing referral pathway for patients with PD and cognitive impairment to improve speed of access to assessment and management, reduce unnecessary duplication (and therefore costs) and improve patient experience.

## 2. Materials and Methods

### 2.1. Context

**Figure 1:**
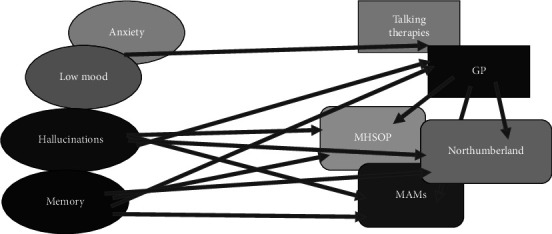
Existing referral pathway.

Our existing referral pathway was complex and depended on a number of considerations including patients' postal address, GP address, clinician and patient preferences (Figure 1). Patients experiencing hallucinations or cognitive impairment could be referred to a specialist mental health team via their GP or directly from our PD team. Depending on geography, this would be one of three different services—North Tyneside Mental Health Service for Older People (MHSOP), Newcastle Memory Assessment Service (MAMs) or Northumberland Memory Service. The majority of our referrals went to MHSOP—this would lead to an initial assessment from a memory specialist nurse followed by a consultant psychiatrist diagnostic appointment. Patients would then either remain under review by the mental health team or be discharged. They would continue to see the PD MDT as normal. The PD MDT and MHSOP did not have access to a shared patient record, and the PD team was not consistently informed about psychiatric diagnosis or treatment.

### 2.2. Intervention

**Figure 2:**
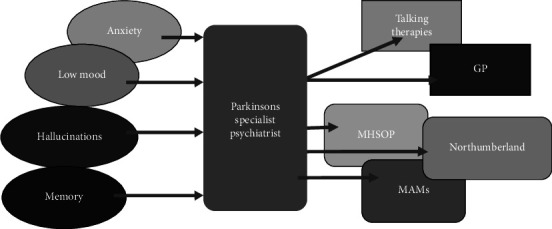
New referral pathway.

We integrated one session per week of input from a consultant PD specialist psychiatrist (PDSP) into our existing Parkinson's MDT service. The PDSP reviewed patients in the same location as the rest of the PD MDT. Referrals to the PDSP could be made by any member of the MDT in relation to any mental health symptom including cognitive impairment, and their proximity meant that informal queries from the MDT could be made during clinical sessions. Following initial review, our PDSP determined whether diagnosis and treatment of mental health symptoms could continue via our MDT or make an onward referral if more specialist psychiatric assessment and support was required (Figure 2). This might include patients with an uncertain diagnosis, patients who needed regular review with a community psychiatric nurse (CPN), or for family supporter behavioural symptoms via a service such as the Admiral Nursing team [13].

### 2.3. Assessment of Impact—Data Collection and Analysis

Following implementation of the new referral pathway, we collected data about referrals of patients with PD and psychiatric symptoms to our PDSP over a period of 1 year A matched pre- and postcomparison was not possible as we were unable to obtain a retrospective sample of patients with PD and psychiatric symptoms prior to initiating our service change—our electronic clinical records did not allow sufficiently granular coding of information to identify samples of patients with particular psychiatric symptoms or diagnoses.

To evaluate the impact of the new pathway, we considered:

• Referral symptoms
• Diagnostic trajectories of patients with PD and cognitive impairment—time to review, time to diagnosis, treatment and follow-up
• Estimated cost savings of an initial review by our PDSP (by comparison with the former referral pathway to MHSOP)
• Case studies of patients with PD and cognitive impairment before and after service change

Patients referred to our PDSP over 12 months were identified from clinic lists held on hospital administrative systems. The patients' hospital codes were used to review clinical information available from various digital records including clinic letters, clinical noting and SystmOne. The following data were entered into a spreadsheet and analysed using simple descriptive statistics.

For patients with cognitive impairment, estimated cost savings were calculated by comparing the costs of assessment and management steps in the previous referral pathway to the costs of the new pathway. The trust business units supplied the following costs for patients referred using the previous pathway. This pathway required a member of the PD MDT to send a paper referral to MHSOP to generate an appointment, followed by an initial specialist nursing assessment, followed by a consultant psychiatrist diagnostic assessment (Table 1). The new pathway with direct referral to the PDSP did not require the administrative cost or specialist nursing assessment.

Analysis was made more complex by a delay to agreement of the new referral pathway. This meant that for 3 months patients seen by our PDSP also had to be referred using the previous pathway, leading to duplication of assessment and additional costs. We therefore performed a secondary analysis of 9 months of referrals once the new pathway was confirmed.

As a service evaluation of routinely collected clinical data, formal ethical approval was not required but Caldicott approval was confirmed.

## 3. Results

**Figure 3:**
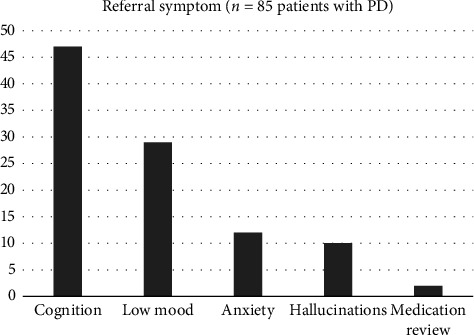
Symptoms prompting referral of patients with PD to PDSP.

Over 12 months, 85 patients were referred to and assessed by our PDSP. The most common referral symptom was cognitive impairment (55%), followed by low mood (34%), anxiety (14%) and hallucinations (12%) (Figure 3). Median time from referral to review was 1 month.

### 3.1. Diagnoses and Outcomes

**Figure 4:**
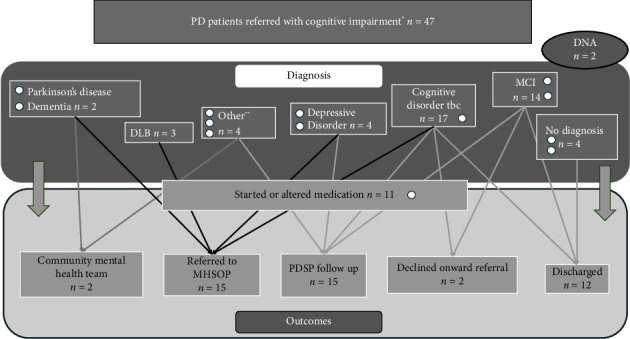
Diagnoses and outcomes for 47 patients referred with cognitive impairment. ^∗^With or without low mood. ^∗∗^Anxiety disorder *n* = 2, adjustment disorder *n* = 1, cognitive disorder due to schizophrenia *n* = 1. DLB: dementia with Lewy bodies. DNA: did not attend. MCI: mild cognitive impairment. MHSOP: Mental Health Service for Older People. PDSP: Parkinson's disease specialist psychiatrist.

Thirty patients referred with anxiety or low mood (without cognitive impairment) received diagnoses including depressive disorders and adjustment disorders or were confirmed to have no formal psychiatric diagnosis. Forty-seven patients with cognitive impairment (with or without co-existing low mood) were seen and received a range of diagnoses including mild cognitive impairment (MCI), depressive disorder and DLB (Figure 4). Eleven were commenced on medication by the PDSP.

Outcomes for patients with cognitive impairment included discharge (*n* = 12), referral to another mental health team for further MDT evaluation (15), or ongoing follow-up with the PDSP (15). Of the 47 patients referred with cognitive impairment, initial review by our PDSP meant that 29 did not need onward referral to a mental health service (as per our previous pathway).

### 3.2. Cost Savings

Over a period of 12 months, the estimated cost saving attributed to the new pathway was £365.75. For the first 3 months, until the new pathway was agreed and finalised, patients with PD and psychiatric symptoms were referred to the PDSP and then on to MHSOP as per the existing pathway which led to duplication of consultant time and a resultant cost increase of approximately £129.08 per patient. Excluding costs from these 3 months and considering the 9 months post implementation of the new pathway, estimated cost savings were £1016.36.

The greatest cost savings were made for patients diagnosed with MCI. Via the new pathway, patients required only one clinical contact with the PDSP rather than a specialist nursing assessment followed by a consultant diagnostic review—the cost saving per patient was an estimated £124.49, and £1328.79 for the cohort of 14 patients with MCI.

### 3.3. Case Studies

Prior to implementation of the new direct pathway to our PDSP, MDT members were sometimes uncertain as to the best method of referral for assessment and treatment of patients with PD and psychiatric symptoms. PD specialist nurses did not always feel confident enough to initiate referral to another mental health service, and some referrals occurred via general practice, adding another clinical contact (Appendix A). In this example, diagnosis took 7 months and required contact with four different HCPs.

In contrast, the option of access to a PDSP for informal advice and discussion prior to referral meant that MDT members could be confident that the referral was appropriate and contained all the necessary information (Appendix B). Diagnosis in this case took 3 months and required contact with two HCPs.

## 4. Discussion

Integration of a PDSP into our MDT introduced a more streamlined diagnostic pathway for patients with PD and psychiatric symptoms. Prior to the new pathway, referrals were not consistent and varied according to predominant symptoms and individual MDT members' level of comfort in managing those symptoms or signposting the patient to another service. Integration of the PDSP provided the MDT with a consistent source of advice and minimised inappropriate referrals. Anecdotally, patients and their families expressed a preference for being seen within our MDT rather than having to be referred to a different service.

Patients with PD and psychiatric symptoms could be referred and reviewed with a median time of 1 month. The majority of patients were assessed, treated and reviewed by our PDSP without requiring onward referral to another mental health service. For the minority who did need referral to another specialist psychiatric service, initial assessment by our PDSP meant that they could be referred directly to the most appropriate team for ongoing treatment and monitoring, for example, a community mental health nursing team.

The rationalisation afforded by streamlined referral to our PDSP for patients with cognitive impairment led to quicker assessment and likely cost savings. The period of 3 month overlap between the old and new pathways did lead to an initial estimated cost increase, but exclusion of this period suggested cost savings of more than one thousand pounds over 9 months. An unexpected finding was that the greatest estimated cost savings related to patients diagnosed with MCI. For this group, a single contact with our PDSP meant assessment, diagnosis, and explanation at a single attendance rather than the previous pathway of referral, initial specialist nursing assessment and subsequent consultant diagnostic assessment. Prompt recognition of MCI in PD has been highlighted as a clinical priority in order to monitor and address cognitive decline in cohorts of patients with PD [14].

The key limitation of our analysis relates to the difficulties of obtaining a pre- and post-implementation matched sample of patients with PD and psychiatric symptoms. Therefore, we cannot make a direct comparison of time to review, time to diagnosis or actual costs. In the context of a local quality improvement project a controlled trial design to evaluate causality was not feasible. Our cost saving estimates are based on the standard tariffs attached to generic clinical activities—the reduction in clinical contacts afforded by direct referral to our PDSP means that is it highly likely that the new pathway is more efficient than the previous.

A further limitation is lack of certainty as to patient outcomes—timely assessment and diagnosis is beneficial, but we cannot claim that our new pathway led to better quality of care. Rather than directly measuring clinical effectiveness, our analysis is based on process efficiency. To better determine the direct impact of different service models for patients with PD and psychiatric symptoms, future studies should consider a controlled trial design with comparative longitudinal measurement of outcomes such as patient QoL, carer stress or rates of admissions to hospital. Qualitative research with patients with PD and cognitive impairment suggests that existing models of care are disjointed and under-resourced [15]—further exploration of how patient and carer views on how MDT services can be adapted to better meet their needs would also be of benefit.

Despite these limitations, integration of a PDSP into an MDT is a change that could be adopted by other PD services nationally and internationally. Our analysis provides some evidence of efficiency savings and examples of patients who are likely to have benefitted from more rapid assessment, diagnosis and treatment. In keeping with quality improvement methodology, the optimum service model for patients with PD and psychiatric symptoms is likely to vary according to local factors including demographics, existing health service governance and resource availability.

## 5. Conclusions

Psychiatric symptoms in patients with PD are common, with a significant impact on QoL, but there is no evidence as to the best model of care for these patients. We demonstrated that integration of a PDSP into an MDT led to a streamlined pathway to assessment, diagnosis, and treatment, with estimated cost savings. This is a model that could be adopted elsewhere to allow MDTs caring for patients with PD to be able to offer more holistic care for complex patients.

## Figures and Tables

**Table 1:** Costs associated with previous referral pathway.

Previous pathway steps	Costs
Administration of referral to MHSOP	£15.31
Specialist nursing assessment	£109.18
Consultant diagnostic review	£129.08
Total	£253.57

## Data Availability

Data sharing is not applicable to this article as no new data were created or analyzed in this study.

## Appendix A: Existing Referral Pathway

March 2023—Male patient in their 80s, diagnosed with PD 10 years ago, living independently. At routine PD review MDT note that he is starting to struggle to manage appointments and family are concerned that he is not taking medication as prescribed.

April 2023—PD specialist nursing review to perform cognitive assessment (MOCA 15/30) followed by a request to GP to refer to MHSOP. Initial referral rejected as up to date bloods not available.

July 2023—MHSOP Specialist Nursing assessment and request for brain imaging.

September 2023—Consultant review and diagnosis of PDD. Rivastigmine commenced.

## Appendix B: New Referral Pathway

March 2023—Male patient in their 80s, diagnosed with PD 10 years ago, living independently. At routine PD review MDT note that he is starting to struggle to manage appointments and family are concerned that he is not taking medication as prescribed. Discussed with PDSP who confirmed the referral was appropriate and that up-to-date bloods were available.

April 2023—PDSP review. Likely diagnosis of PDD, imaging requested.

June 2023—PDSP follow-up. Confirmed PDD and commenced Rivastigmine.
